# The right to science and gender inequalities

**DOI:** 10.3389/fsoc.2023.1285641

**Published:** 2023-11-17

**Authors:** Yvonne Donders

**Affiliations:** Faculty of Law, Department of International and European Public Law, University of Amsterdam, Amsterdam, Netherlands

**Keywords:** right to science, gender inequalities, women's rights, UNESCO, gender bias against women, human rights, economic, social and cultural rights, right to education

## Introduction

The right to science was included in the Universal Declaration of Human Rights 75 years ago.^1^ A very influential person in the drafting of the Universal Declaration was a woman, Eleanor Roosevelt. The position of women in the UDHR is, however, rather secondary. The UDHR speaks of all members of the human family, but its text is rather male oriented. Moreover, despite the fact that equality and non-discrimination are at the heart of the UDHR, women still do not enjoy human rights equally with men. The same is true for the right to science.

It is uncontested that deep inequalities between women and men persist in the field of education, sciences and research. Women remain underrepresented and/or disadvantaged in access to scientific education and opportunities to have a career in academia.^2^ Furthermore, women suffer from lack of access to scientific applications and scientific applications and technologies may be gender biased and not sensitive to the particularities and needs of women.^3^

This inequality is persistent, “[…] even in countries with relatively long histories of formal and legal equality”.^4^ This implies that more subtle and underlying factors play a role, such as gender stereotypes and biases. How ineradicable is the inequality between women and men in sciences and research and what does the right to science as a human right have to offer in response to sex and gender inequalities? This opinion focuses on the right to science as included in Article 15(1)b of the International Covenant on Economic, Social and Cultural Rights and in the UNESCO Recommendation on Science and Scientific Researchers. It argues that the right to science is an important normative tool that should promote and facilitate structural changes that can help to overcome gender-based barriers and ensure that women enjoy the right to science equally with men.

## Persistent gender inequalities in sciences and research

One of the core principles of all human rights treaties is the obligation of States to ensure equal enjoyment of human rights and to prevent and put an end to all forms of discrimination.^5^ There are different forms of discrimination, including direct, indirect and structural discrimination, that should be prevented and eliminated.^6^ All three can cause or sustain the disadvantaged position of women in sciences and research.

Direct discrimination in relation to sciences occurs when women and girls are formally and thereby explicitly excluded from participating, accessing, or contributing to education, sciences and research. Examples are States that have laws and policies that prohibit women from accessing and participating in education and sciences. The recent measures taken by the Taliban regime in Afghanistan, where girls and women were officially expelled from universities and banned from schools, is a striking and sad example.

Indirect discrimination of women as regards their right to science can occur when rules and procedures are seemingly neutral, but in practice have a specific disadvantage for women. For instance, there can be certain requirements for academics to be eligible for promotion or for a leadership position, that may apply to all, but that can be more difficult to meet for women, because of structural inequalities in the right to education and in the right to participate in public life. A concrete example of such a requirement can be that a substantive part of the scientific education and experience has to be acquired in renowned academic institutions abroad. This may constitute a barrier especially for women who want or need to be more confined to their country, region, home or family, or for whom an extensive stay abroad cannot be combined with caretaking tasks (UNESCO Science Report, 2021).

Systemic and structural discrimination may overlap with indirect discrimination. Systemic but subtle mechanisms or professional practices may discourage women's participation in sciences and research and contribute to their underrepresentation. For instance, there can be a culture of fierce academic competition for research funding or grants, whereby the competition for such grants is not gender neutral. For example, research has shown that women have less chance of having their work published in peer reviewed international journals (Squazzoni et al., 2021; Kern-Goldberger et al., 2022; Bornmann et al., 2023). A well-known structural barrier for many women is that a successful career in scientific research is only possible if someone invests and devotes large amounts of time to doing research. This may be difficult or impossible for women in periods of their life that are also crucial for founding a family. For men it is easier to postpone becoming a parent until a later age than it is for women.^7^

States have clear obligations to promote and to put into effect formal (de jure), substantive (de facto) and transformative (structural) equality of women, responding to direct, indirect and structural discrimination.^8^ Achieving formal, substantive and structural equality may also imply taking (temporary) special measures to promote and protect inclusion and participation of women.^9^ According to the UN Committee on Economic, Social and Cultural Rights (CESCR), ‘[…] the principle of equality will sometimes require that States parties take measures in favor of women in order to attenuate or suppress conditions that perpetuate discrimination. As long as these measures are necessary to redress de facto discrimination and are terminated when de facto equality is achieved, such differentiation is legitimate.'^10^

## What can the right to science offer?

The right to science covers various aspects and dimensions, including access, participation, contribution and enjoying the benefits, as well as protection against harmful science.^11^ All these dimensions have gender dimensions that seem to sustain inequalities between women and men in relation to sciences and research. The Committee on Economic, Social and Cultural Rights, which is the independent body monitoring the implementation of the treaty by the States parties, has elaborated on the normative content of the right to science, including its gender dimensions.

In its General Comment on science and economic, social and cultural rights the CESCR explicitly urged States parties to take measures to tackle these gender dimensions. It firstly expressed that States must “[…] immediately eliminate barriers that affect girls' and women's access to quality scientific education and careers'. States must also take steps” […] to ensure women's substantive equality in access to scientific education and careers by, for example, raising public awareness in order to eliminate stereotypes that exclude women from science or adopting policies for both men and women to balance domestic life with scientific careers'. It also maintained that ”[…] temporary special measures, such as quotas for women in scientific education, might be necessary” in order to advance more quickly toward substantive equality in the enjoyment of the right to science.^12^

The CESCR furthermore addressed the gender dimensions of scientific research by stating that “[a] gender-sensitive approach is not a luxury for scientific research, but a crucial tool in order to ensure that scientific progress and new technologies adequately take into account the characteristics and needs of women and girls”.^13^ A gender sensitive approach should be part of all stages of the process of research, from the choice of subjects and the design of methodologies up to the evaluation of its applications and impacts. The CESCR also urges States parties to make decisions concerning funding or general policies in a (more) gender-sensitive manner^13^.

The CESCR formulates a wide palette of State obligations to respect, protect and fulfill the right to science, including the equal enjoyment of this right by women and men.^14^ Moreover, it named the identification and elimination of all laws, policies, practices, prejudices or stereotypes that undermine women's and girls' participation in science as a so-called core obligation.^15^ A core obligation is the minimum essential level of a right that all States parties, irrespective of their political, economic or social situation, should immediately guarantee.^16^

The Member States of UNESCO have adopted various instruments on science and education, and for many years “gender equality” is one of the key priorities of UNESCO.^17^ The UNESCO Recommendation on Science and Scientific Researchers^18^ addresses many of the gender inequality issues related to sciences and research as outlined above.

The promotion of equality and non-discrimination is explicitly addressed in relation to education and training of scientific researchers, an area in which States are recommended to take measures to abolish inequalities in opportunities. States are recommended to guarantee equal opportunities in education and training needed to qualify for research careers, as well as for those qualified equal access to available employment in scientific research.^19^

States are further recommended, “[…] in order to remediate past inequalities and patterns of exclusion”, to “[…] actively encourage women…to consider careers in sciences, and endeavor to eliminate biases against women […] in work environments and appraisal”^20^ and they are encouraged to “[…] ensure that scientific researchers enjoy equitable conditions of work, recruitment and promotion, appraisal, training and pay without discrimination on the basis of […] sex [and] gender […]”.^21^

Appraisal is an important area where gender-based differences can play a role. It is therefore recommended that States should “[…] design and establish appropriate appraisal systems for independent, transparent, gender-sensitive and tier-based performance evaluation”.^22^ Such systems should take due account of “[…] the difficulty inherent in measuring a performance given the effects of mobility between themes and disciplines […] and the need to appraise all aspects of the individual's performance in context”;^23^ and they should “[…] transparently account for family-care related interruptions of employment and encourage equitable treatment by means of incentives, so that the careers and research of those who take family related leave, including parental leave, are not negatively impacted as a result”.^24^

In short, the right to science, in combination with the general prohibition of all forms of discrimination, clearly implies that women have the right to equally access, participate in and contribute to sciences and research and that States have positive obligations to ensure this right. There is no lack of applicable norms and no lack of clarity on State obligations. These obligations are not merely obligations of conduct, but also of result. It seems that the continuous inequality between women and men in sciences and research is caused and sustained by a lack of effective implementation and monitoring of these norms.

## Discussion

The right to science includes rights to participate in science, to contribute to science, to have access to science and to enjoy the benefits of science. These rights should be equally enjoyed by all, based on ability and competence. In all these areas, however, women are disadvantaged. The underrepresentation of women in sciences and research also has consequences for the applications of science, which can reflect or sustain problematic gender inequalities.

Different forms of discrimination—direct, indirect and structural—negatively affect the enjoyment of the right to science by women. In particular, structural and systematic forms of discrimination caused by persistent gender stereotypes and patterns in societies sustain the subordinate position of women and prevent them from fully participating. Effective participation by women in science education, in sciences and research, as well as in the development of science policies and science agendas, therefore requires formal and substantial equality, but mostly transformative equality and structural changes. To overcome institutional and societal gender-based barriers and ensure that women enjoy the right to science equally with men, special policies and measures are required. The elimination of discrimination, including stereotypes that undermine women's participation in science, is a core obligation that States should respect, protect and fulfill under all circumstances.

The normative framework of the right to science is well-established, with the different human rights treaties and UNESCO instruments. Supported by the general principles of equality and non-discrimination, it is an important right that should promote and protect women's participation in sciences and research, which could also contribute to more gender equality in scientific applications. The international legal instruments on the right to science demonstrate a large degree of consensus among States on the need, at least in theory, to promote gender equality in sciences and research. It does, however, seem to be much more difficult to advance this formal articulation beyond the realm of aspirations. Concrete and effective action is however urgently needed. The right to science exists for 75 years now. It is time to start using it as a normative tool to repair and prevent gender inequalities in sciences and research.

## Author contributions

YD: Writing – original draft.

## Footnotes

^1^Article 27 of the UDHR includes the right of everyone to “share in scientific advancement and its benefits”. The term “right to science” is used for reasons of practicality and familiarity in dominant scholarship on this matter. The right to science is however an umbrella term for a cluster of rights.

^2^Committee on Economic, Social and Cultural Rights, General comment No. 25 (2020) on science and economic, social and cultural rights (article 15 (1) (b), (2), (3) and (4) of the International Covenant on Economic, Social and Cultural Rights), 30 April 2020, UN Doc. E/C.12/GC/25, p. 7, para. 29 (UNESCO, 2017; UNESCO Institute of Statistics, 2019; European Commission, 2021; 108; UNESCO Science Report, 2021).

^3^Committee on Economic, Social and Cultural Rights, *General Comment No. 25 (2020) on science and economic, social and cultural rights (article 15 (1) (b), (2), (3) and (4) of the International Covenant on Economic, Social and Cultural Rights)* (30 April 2020 UN Doc. E/C.12/GC/25), p. 7, para. 30.

^4^*Report of the Special Rapporteur in the field of cultural rights on the enjoyment of cultural rights by women on an equal basis with men* (10 August 2012 UN Doc. A/67/287) p. 15, para. 48.

^5^International Covenant on Economic, Social and Cultural Rights (ICESCR, 1966) Articles 2 and 3; The Convention on the Elimination of all Forms of Discrimination Against Women (CEDAW, 1979) contains many relevant norms, including Articles 1 and 5. This short opinion focuses on the ICESCR, since it contains also the right to science; for elaboration of the CEDAW see the original chapter (Donders, 2023).

^6^Committee on Economic, Social and Cultural Rights, *General Comment No. 16, The equal right of men and women to the enjoyment of all economic, social and cultural rights (art. 3 of the International Covenant on Economic, Social and Cultural Rights)* (UN Doc. E/C.12/2005/4 11 August 2005); CEDAW Committee, *General Recommendation No. 25, on article 4, paragraph 1, of the Convention on the Elimination of All Forms of Discrimination against Women, on temporary special measures* (18 August 2004); CEDAW Committee, *General Recommendation No. 28 on the core obligations of States parties under article 2 of the Convention on the Elimination of All Forms of Discrimination against Women* (UN Doc. CEDAW/C/GC/28 16 December 2010).

^7^A lot has been published on women and their academic careers in relation to motherhood. There is an organization for mothers in science (https://www.mothersinscience.com/) that aims to help an support mothers in STEM research. See, also: Wendy and Ceci (2012), Global Young Academy (2020), Powell (2021), Available here: https://www.americanscientist.org/article/when-scientists-choose-motherhood.

^8^Human Rights Council, *Thematic Study on discrimination in law and practice and how the issue is addressed throughout the United Nations human right's system* (31 August 2010 UN Doc A/HRC/15/40) para 10.

^9^*Convention on the Elimination of All Forms of Discrimination Against Women* (adopted 18 December 1979, entry into force 3 September 1981), Article 4.

^10^Committee on Economic, Social and Cultural Rights, *General Comment No. 16, The equal right of men and women to the enjoyment of all economic, social and cultural rights (art. 3 of the International Covenant on Economic, Social and Cultural Rights)* (11 August 2005 UN Doc. E/C.12/2005/4) p. 4, para. 15.

^11^Article 15(1)b of the International Covenant on Economic, social and Cultural Rights includes the right of everyone to “enjoy the benefits of scientific progress and its applications”. Committee on Economic, Social and Cultural Rights, *General Comment No. 25 (2020) on science and economic, social and cultural rights (article 15 (1) (b), (2), (3) and (4) of the International Covenant on Economic, Social and Cultural Rights)* (30 April 2020 UN Doc. E/C.12/GC/25) p. 4-5, paras. 16-20. The right to science is increasingly unpacked by scholars (Besson, 2015; Donders, 2015; Mann et al., 2020; Boggio, 2021; Porsdam, 2022; Porsdam and Mann, 2022).

^12^Committee on Economic, Social and Cultural Rights, *General Comment No. 25 (2020) on science and economic, social and cultural rights (article 15 (1) (b), (2), (3) and (4) of the International Covenant on Economic, Social and Cultural Rights)* (30 April 2020 UN Doc. E/C.12/GC/25) p. 7, para. 31.

^13^Committee on Economic, Social and Cultural Rights, *General Comment No. 25 (2020) on science and economic, social and cultural rights (article 15 (1) (b), (2), (3) and (4) of the International Covenant on Economic, Social and Cultural Rights)* (30 April 2020 UN Doc. E/C.12/GC/25) p. 7, para. 32.

^14^Committee on Economic, Social and Cultural Rights, *General Comment No. 25 (2020) on science and economic, social and cultural rights (article 15 (1) (b), (2), (3) and (4) of the International Covenant on Economic, Social and Cultural Rights)* (30 April 2020 UN Doc. E/C.12/GC/25) pp. 9-10, paras. 43-50.

^15^Committee on Economic, Social and Cultural Rights, *General Comment No. 25 (2020) on science and economic, social and cultural rights (article 15 (1) (b), (2), (3) and (4) of the International Covenant on Economic, Social and Cultural Rights)* (30 April 2020 UN Doc. E/C.12/GC/25) pp. 10-11, paras. 51 and 52.

^16^Committee on Economic, Social and Cultural Rights, *General Comment No. 3: The Nature of States Parties' Obligations (Art. 2, Para. 1, of the Covenant)* (14 December 1990 UN Doc. E/1991/23) para. 10.

^17^See, on the work of UNESCO in relation to right to science: Donders and Tararas (2021).

^18^*UNESCO Recommendation on Science and Scientific Researchers* (Paris November 2017 Doc. 39 C/Resolution 85). This Recommendation superseded the *UNESCO Recommendation on the Status of Scientific Researchers* (Paris 1974 Doc. 18 C/Res.40).

^19^*UNESCO Recommendation on Science and Scientific Researchers* (Paris November 2017 Doc. 39 C/Resolution 85) para. 13(a) and 13(b).

^20^*UNESCO Recommendation on Science and Scientific Researchers* (Paris November 2017 Doc. 39 C/Resolution 85) para. 13(c).

^21^*UNESCO Recommendation on Science and Scientific Researchers* (Paris November 2017 Doc. 39 C/Resolution 85) para. 24(b).

^22^*UNESCO Recommendation on Science and Scientific Researchers* (Paris November 2017 Doc. 39 C/Resolution 85) para. 34.

^23^*UNESCO Recommendation on Science and Scientific Researchers* (Paris November 2017 Doc. 39 C/Resolution 85) para. 34 (b).

^24^*UNESCO Recommendation on Science and Scientific Researchers* (Paris November 2017 Doc. 39 C/Resolution 85) para. 34 (d).
